# Dibromidobis[2-(dicyclo­hexyl­phosphan­yl)­biphenyl-κ*P*]palladium(II)

**DOI:** 10.1107/S1600536808030845

**Published:** 2008-09-30

**Authors:** Chen Xu, Ying-Fei Li, Zhi-Qiang Wang, Fei-Fei Cen, Yu-Qing Zhang

**Affiliations:** a College of Chemistry and Chemical Engineering, Luoyang Normal University, Luoyang 471022, People’s Republic of China; b Chemical Engineering and Pharmaceutics School, Henan University of Science and Technology, Luoyang 471003, People’s Republic of China

## Abstract

The title compound, [PdBr_2_(C_24_H_31_P)_2_], has a distorted *trans* square-planar coordination of the Pd atom, which occupies an inversion centre. The most important bond distances include Pd—P of 2.380 (2) Å and Pd—Br of 2.515 (2) Å. Weak inter­molecular π–π inter­actions between the benzene rings of adjacent mol­ecules [centroid–centroid distance = 3.949 (6) Å] are present *via* crystallographic inversion centres, resulting in a one-dimensional supra­molecular architecture.

## Related literature

For related literature, see: Barder *et al.* (2005 ▶); Christmann *et al.* (2006 ▶); Stark & Whitmire (1997 ▶); Tomori *et al.* (2000 ▶); Tsuji (1995 ▶); Xu *et al.* (2007 ▶).
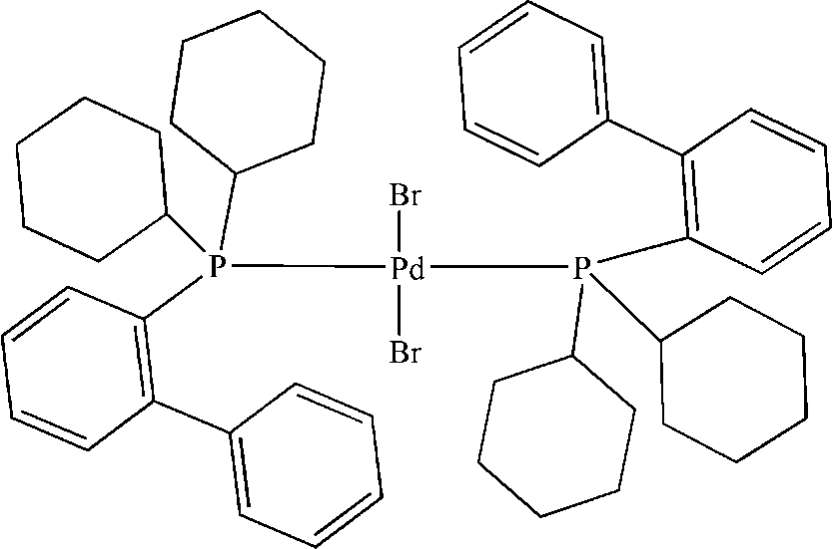


## Experimental

### 

#### Crystal data

[PdBr_2_(C_24_H_31_P)_2_]
*M* *_r_* = 967.14
Triclinic, 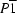

*a* = 9.817 (8) Å
*b* = 9.827 (8) Å
*c* = 11.957 (10) Å
α = 91.582 (11)°
β = 108.822 (10)°
γ = 103.713 (10)°
*V* = 1053.9 (15) Å^3^
*Z* = 1
Mo *K*α radiation
μ = 2.44 mm^−1^
*T* = 291 (2) K
0.14 × 0.10 × 0.09 mm

#### Data collection

Bruker SMART APEX CCD diffractometer
Absorption correction: multi-scan (*SADABS*; Sheldrick, 1996 ▶) *T*_min_ = 0.723, *T*_max_ = 0.803
7316 measured reflections
3811 independent reflections
2810 reflections with *I* > 2σ(*I*)
*R*_int_ = 0.038

#### Refinement

*R*[*F* ^2^ > 2σ(*F* ^2^)] = 0.066
*wR*(*F* ^2^) = 0.180
*S* = 1.10
3811 reflections
241 parameters
H-atom parameters constrained
Δρ_max_ = 0.71 e Å^−3^
Δρ_min_ = −1.42 e Å^−3^

### 

Data collection: *SMART* (Bruker, 2004 ▶); cell refinement: *SAINT* (Bruker, 2004 ▶); data reduction: *SAINT*; program(s) used to solve structure: *SHELXS97* (Sheldrick, 2008 ▶); program(s) used to refine structure: *SHELXL97* (Sheldrick, 2008 ▶); molecular graphics: *SHELXTL* (Sheldrick, 2008 ▶); software used to prepare material for publication: *PLATON* (Spek, 2003 ▶) and *SHELXTL*.

## Supplementary Material

Crystal structure: contains datablocks global, I. DOI: 10.1107/S1600536808030845/si2114sup1.cif

Structure factors: contains datablocks I. DOI: 10.1107/S1600536808030845/si2114Isup2.hkl

Additional supplementary materials:  crystallographic information; 3D view; checkCIF report

## Figures and Tables

**Fig. 1.**
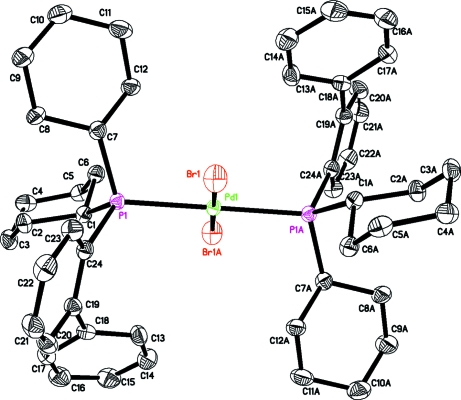
The molecular structure of the title compound with displacement ellipsoids of the non-hydrogen atoms drawn at the 30% probability level. Inversion related atoms are labelled with an A.(Symmetry code: 2 - x, 1 - y, 1 - z).

**Fig. 2.**
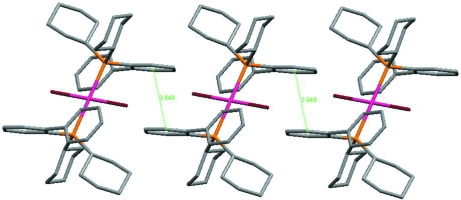
Partial view of the crystal packing showing the formation of the chain motif of molecules formed by the intermolecular π···π interactions, extending along the a axis.

## supplementary crystallographic information

### Comment

Phosphine complexes of palladium have widely been used as catalysts for various reactions (Tsuji, 1995). These complexes are easily prepared from palladium(II) salts and an excess of phosphine ligands. Among them, monophosphinobiaryl complexes of palladium are one of the most important ones (Barder *et al.*, 2005; Christmann *et al.*, 2006; Xu *et al.*, 2007).

The title complex has crystallographic inversion symmetry C_i_ (Fig.1). The Pd atom is in a square-planar environment, while the *trans* 2-(Dicyclohexylphosphanyl)biphenyl ligands are in an eclipsed conformation. The dihedral angles of the benzene rings are 60.8 (2)°. The Pd—P [2.380 (2) Å] and Pd—Br [2.515 (5) Å] bond lengths are longer than the related triphenylphosphine complex of palladium [2.336 (2)Å and 2.4169 (13) Å](Stark & Whitmire, 1997) possibly due to the steric bulk of the ligand. Weak intermolecular π···π interactions between the benzene rings C19 - C24 (Cg4) of inversion related adjacent molecules [centroid-centroid distance Cg4···Cg4^ii^ is 3.949 (6) Å, the perpendicular distance Cg4 on ring Cg4^ii^ is 3.582 Å, and the slippage is 1.663 Å, symmetry code ii = 1 - *x*, 1 - *y*, 1 - *z*] were calculated for the structure of the title complex with the programme PLATON (Spek, 2003), resulting in a one-dimensional supramolecular architecture.

### Experimental

2-(Dicyclohexylphosphanyl)biphenyl was prepared as described in the literature (Tomori *et al.*, 2000). A solution of PdBr_2_(PhCN)_2_ (1 mmol) and 2-(Dicyclohexylphosphanyl)biphenyl (2 mmol) in dry benzene (5 ml) was stirred for 1 day, removal of solvent resulted in a yellow powder that was recrystallized from dichloromethane-petroleum ether solution at room temperature to give the desired product as yellow crystals suitable for single-crystal X-ray diffraction.

### Refinement

H atoms were placed in calculated positions (C*sp^2^*—H = 0.93 Å, C*sp^3^*—H = 0.97 -0.98 Å) and refined as riding on their carriers with isotropic displacement parameters *U*_iso_(H) = 1.2 times *U*_eq_(C).

### Crystal data

**Table:**

[PdBr_2_(C_24_H_31_P)_2_]	*Z* = 1
*M**_r_* = 967.14	*F*(000) = 496
Triclinic, *P*1	*D*_x_ = 1.524 Mg m^−^^3^
*a* = 9.817 (8) Å	Mo *K*α radiation, λ = 0.71073 Å
*b* = 9.827 (8) Å	Cell parameters from 1503 reflections
*c* = 11.957 (10) Å	θ = 2.4–21.7°
α = 91.582 (11)°	µ = 2.45 mm^−^^1^
β = 108.822 (10)°	*T* = 291 K
γ = 103.713 (10)°	Block, yellow
*V* = 1053.9 (15) Å^3^	0.14 × 0.10 × 0.09 mm

### Data collection

**Table:**

Bruker SMART APEX CCD diffractometer	3811 independent reflections
Radiation source: fine-focus sealed tube	2810 reflections with *I* > 2σ(*I*)
graphite	*R*_int_ = 0.039
φ and ω scans	θ_max_ = 25.5°, θ_min_ = 2.4°
Absorption correction: multi-scan (SADABS; Sheldrick, 1996)	*h* = −11→11
*T*_min_ = 0.723, *T*_max_ = 0.803	*k* = −11→11
7316 measured reflections	*l* = −14→14

### Refinement

**Table:**

Refinement on *F*^2^	Primary atom site location: structure-invariant direct methods
Least-squares matrix: full	Secondary atom site location: difference Fourier map
*R*[*F*^2^ > 2σ(*F*^2^)] = 0.066	Hydrogen site location: inferred from neighbouring sites
*wR*(*F*^2^) = 0.180	H-atom parameters constrained
*S* = 1.10	*w* = 1/[σ^2^(*F*_o_^2^) + (0.0609*P*)^2^ + 7.3363*P*] where *P* = (*F*_o_^2^ + 2*F*_c_^2^)/3
3811 reflections	(Δ/σ)_max_ < 0.001
241 parameters	Δρ_max_ = 0.71 e Å^−^^3^
0 restraints	Δρ_min_ = −1.42 e Å^−^^3^

### Special details

**Table:**

Geometry. All e.s.d.'s (except the e.s.d. in the dihedral angle between two l.s. planes)are estimated using the full covariance matrix. The cell e.s.d.'s are takeninto account individually in the estimation of e.s.d.'s in distances, anglesand torsion angles; correlations between e.s.d.'s in cell parameters are onlyused when they are defined by crystal symmetry. An approximate (isotropic)treatment of cell e.s.d.'s is used for estimating e.s.d.'s involving l.s. planes.
Refinement. Refinement of *F*^2^ against ALL reflections. The weighted *R*-factor *wR* and goodness of fit *S* are based on *F*^2^, conventional *R*-factors *R* are based on *F*, with *F* set to zero for negative *F*^2^. The threshold expression of *F*^2^ > σ(*F*^2^) is used only for calculating *R*-factors(gt) *etc*. and is not relevant to the choice of reflections for refinement. *R*-factors based on *F*^2^ are statistically about twice as large as those based on *F*, and *R*- factors based on ALL data will be even larger.

### Fractional atomic coordinates and isotropic or equivalent isotropic displacement parameters (Å^2^)

**Table:**

	*x*	*y*	*z*	*U*_iso_*/*U*_eq_	
Pd1	1.0000	0.5000	0.5000	0.0252 (2)	
Br1	0.96825 (13)	0.71510 (13)	0.59715 (10)	0.0612 (4)	
P1	0.8353 (2)	0.5540 (2)	0.32229 (17)	0.0267 (5)	
C1	0.7959 (9)	0.4354 (9)	0.1852 (7)	0.0306 (18)	
H1	0.7703	0.3392	0.2060	0.037*	
C2	0.6642 (9)	0.4454 (10)	0.0766 (7)	0.038 (2)	
H2A	0.5763	0.4378	0.0990	0.045*	
H2B	0.6870	0.5358	0.0468	0.045*	
C3	0.6343 (10)	0.3264 (10)	−0.0199 (8)	0.044 (2)	
H3A	0.6021	0.2366	0.0078	0.053*	
H3B	0.5546	0.3355	−0.0903	0.053*	
C4	0.7702 (10)	0.3287 (10)	−0.0518 (8)	0.045 (2)	
H4A	0.7945	0.4131	−0.0893	0.054*	
H4B	0.7488	0.2479	−0.1090	0.054*	
C5	0.9032 (10)	0.3258 (9)	0.0551 (8)	0.038 (2)	
H5A	0.9903	0.3344	0.0315	0.046*	
H5B	0.8843	0.2369	0.0879	0.046*	
C6	0.9318 (9)	0.4476 (9)	0.1490 (7)	0.0362 (19)	
H6A	1.0167	0.4456	0.2180	0.043*	
H6B	0.9546	0.5366	0.1171	0.043*	
C7	0.9148 (9)	0.7385 (9)	0.2981 (7)	0.0330 (18)	
H7	0.9069	0.7981	0.3616	0.040*	
C8	0.8334 (10)	0.7900 (9)	0.1839 (8)	0.044 (2)	
H8A	0.8407	0.7375	0.1171	0.052*	
H8B	0.7288	0.7724	0.1751	0.052*	
C9	0.8963 (11)	0.9454 (10)	0.1819 (10)	0.056 (3)	
H9A	0.8471	0.9723	0.1048	0.067*	
H9B	0.8761	0.9987	0.2415	0.067*	
C10	1.0617 (11)	0.9819 (11)	0.2059 (10)	0.057 (3)	
H10A	1.0808	0.9410	0.1396	0.068*	
H10B	1.0993	1.0835	0.2124	0.068*	
C11	1.1426 (10)	0.9292 (10)	0.3177 (10)	0.054 (3)	
H11A	1.1345	0.9794	0.3853	0.064*	
H11B	1.2474	0.9482	0.3268	0.064*	
C12	1.0801 (9)	0.7721 (9)	0.3166 (8)	0.039 (2)	
H12A	1.1322	0.7419	0.3914	0.047*	
H12B	1.0957	0.7210	0.2531	0.047*	
C13	0.6373 (9)	0.2210 (10)	0.3590 (8)	0.043 (2)	
H13	0.7063	0.2713	0.4296	0.052*	
C14	0.6340 (11)	0.0838 (10)	0.3309 (9)	0.048 (2)	
H14	0.7007	0.0422	0.3832	0.058*	
C15	0.5362 (12)	0.0082 (11)	0.2291 (10)	0.055 (3)	
H15	0.5373	−0.0841	0.2107	0.066*	
C16	0.4362 (12)	0.0670 (11)	0.1533 (9)	0.056 (3)	
H16	0.3689	0.0148	0.0829	0.067*	
C17	0.4337 (10)	0.2035 (10)	0.1801 (8)	0.047 (2)	
H17	0.3618	0.2412	0.1292	0.056*	
C18	0.5374 (9)	0.2858 (9)	0.2823 (7)	0.0338 (19)	
C19	0.5333 (9)	0.4319 (9)	0.3139 (7)	0.0324 (18)	
C20	0.4018 (9)	0.4472 (10)	0.3274 (8)	0.043 (2)	
H20	0.3219	0.3678	0.3114	0.051*	
C21	0.3852 (10)	0.5738 (11)	0.3630 (8)	0.047 (2)	
H21	0.2967	0.5795	0.3733	0.056*	
C22	0.4983 (11)	0.6902 (11)	0.3831 (8)	0.046 (2)	
H22	0.4871	0.7770	0.4056	0.056*	
C23	0.6325 (10)	0.6814 (10)	0.3703 (8)	0.040 (2)	
H23	0.7104	0.7626	0.3862	0.048*	
C24	0.6521 (9)	0.5536 (9)	0.3340 (7)	0.0306 (18)	

### Atomic displacement parameters (Å^2^)

**Table:**

	*U*^11^	*U*^22^	*U*^33^	*U*^12^	*U*^13^	*U*^23^
Pd1	0.0260 (5)	0.0262 (5)	0.0248 (5)	0.0099 (4)	0.0083 (3)	0.0043 (3)
Br1	0.0626 (7)	0.0682 (8)	0.0511 (7)	0.0210 (6)	0.0141 (5)	0.0072 (5)
P1	0.0255 (10)	0.0281 (11)	0.0275 (10)	0.0093 (8)	0.0084 (8)	0.0049 (8)
C1	0.032 (4)	0.035 (5)	0.031 (4)	0.014 (4)	0.014 (3)	0.007 (3)
C2	0.032 (4)	0.046 (5)	0.036 (5)	0.015 (4)	0.007 (4)	0.004 (4)
C3	0.043 (5)	0.054 (6)	0.029 (5)	0.009 (4)	0.007 (4)	0.000 (4)
C4	0.051 (6)	0.045 (6)	0.036 (5)	−0.001 (4)	0.021 (4)	−0.005 (4)
C5	0.047 (5)	0.036 (5)	0.043 (5)	0.015 (4)	0.028 (4)	0.004 (4)
C6	0.038 (5)	0.038 (5)	0.034 (4)	0.012 (4)	0.013 (4)	0.005 (4)
C7	0.032 (4)	0.035 (5)	0.036 (5)	0.014 (4)	0.012 (4)	0.007 (4)
C8	0.040 (5)	0.034 (5)	0.056 (6)	0.010 (4)	0.013 (4)	0.020 (4)
C9	0.047 (6)	0.039 (6)	0.077 (7)	0.012 (5)	0.013 (5)	0.024 (5)
C10	0.056 (6)	0.041 (6)	0.072 (7)	0.007 (5)	0.023 (6)	0.019 (5)
C11	0.033 (5)	0.050 (6)	0.073 (7)	0.002 (4)	0.019 (5)	0.013 (5)
C12	0.031 (4)	0.037 (5)	0.051 (5)	0.010 (4)	0.015 (4)	0.014 (4)
C13	0.031 (5)	0.059 (6)	0.039 (5)	0.016 (4)	0.008 (4)	0.010 (4)
C14	0.052 (6)	0.037 (6)	0.059 (6)	0.020 (5)	0.017 (5)	0.013 (5)
C15	0.059 (6)	0.032 (6)	0.068 (7)	0.005 (5)	0.021 (6)	−0.002 (5)
C16	0.055 (6)	0.046 (6)	0.052 (6)	−0.005 (5)	0.011 (5)	−0.004 (5)
C17	0.037 (5)	0.045 (6)	0.044 (5)	0.001 (4)	0.002 (4)	0.007 (4)
C18	0.030 (4)	0.031 (5)	0.039 (5)	0.003 (3)	0.015 (4)	0.003 (4)
C19	0.029 (4)	0.034 (5)	0.035 (4)	0.010 (3)	0.011 (3)	0.004 (4)
C20	0.027 (4)	0.048 (6)	0.052 (6)	0.010 (4)	0.012 (4)	0.007 (4)
C21	0.030 (5)	0.067 (7)	0.053 (6)	0.023 (5)	0.018 (4)	0.009 (5)
C22	0.055 (6)	0.051 (6)	0.051 (6)	0.034 (5)	0.026 (5)	0.012 (5)
C23	0.038 (5)	0.040 (5)	0.047 (5)	0.015 (4)	0.019 (4)	0.005 (4)
C24	0.033 (4)	0.034 (5)	0.029 (4)	0.014 (4)	0.012 (3)	0.006 (3)

### Geometric parameters (Å, °)

**Table:**

Pd1—P1^i^	2.380 (2)	C9—H9B	0.9700
Pd1—P1	2.380 (2)	C10—C11	1.495 (14)
Pd1—Br1^i^	2.515 (2)	C10—H10A	0.9700
Pd1—Br1	2.515 (2)	C10—H10B	0.9700
P1—C24	1.848 (8)	C11—C12	1.518 (13)
P1—C1	1.862 (8)	C11—H11A	0.9700
P1—C7	1.866 (8)	C11—H11B	0.9700
C1—C6	1.510 (11)	C12—H12A	0.9700
C1—C2	1.533 (11)	C12—H12B	0.9700
C1—H1	0.9800	C13—C14	1.371 (13)
C2—C3	1.527 (12)	C13—C18	1.398 (12)
C2—H2A	0.9700	C13—H13	0.9300
C2—H2B	0.9700	C14—C15	1.347 (14)
C3—C4	1.496 (12)	C14—H14	0.9300
C3—H3A	0.9700	C15—C16	1.358 (14)
C3—H3B	0.9700	C15—H15	0.9300
C4—C5	1.511 (12)	C16—C17	1.378 (14)
C4—H4A	0.9700	C16—H16	0.9300
C4—H4B	0.9700	C17—C18	1.391 (12)
C5—C6	1.527 (11)	C17—H17	0.9300
C5—H5A	0.9700	C18—C19	1.488 (12)
C5—H5B	0.9700	C19—C20	1.393 (11)
C6—H6A	0.9700	C19—C24	1.410 (11)
C6—H6B	0.9700	C20—C21	1.367 (13)
C7—C8	1.512 (11)	C20—H20	0.9300
C7—C12	1.518 (11)	C21—C22	1.345 (14)
C7—H7	0.9800	C21—H21	0.9300
C8—C9	1.509 (12)	C22—C23	1.396 (12)
C8—H8A	0.9700	C22—H22	0.9300
C8—H8B	0.9700	C23—C24	1.394 (12)
C9—C10	1.505 (14)	C23—H23	0.9300
C9—H9A	0.9700		
			
P1^i^—Pd1—P1	180.0	C10—C9—C8	111.9 (8)
P1^i^—Pd1—Br1^i^	85.15 (7)	C10—C9—H9A	109.2
P1—Pd1—Br1^i^	94.85 (7)	C8—C9—H9A	109.2
P1^i^—Pd1—Br1	94.85 (7)	C10—C9—H9B	109.2
P1—Pd1—Br1	85.15 (7)	C8—C9—H9B	109.2
Br1^i^—Pd1—Br1	180.000 (2)	H9A—C9—H9B	107.9
C24—P1—C1	106.1 (4)	C11—C10—C9	111.8 (8)
C24—P1—C7	104.9 (4)	C11—C10—H10A	109.2
C1—P1—C7	108.6 (4)	C9—C10—H10A	109.2
C24—P1—Pd1	112.3 (3)	C11—C10—H10B	109.2
C1—P1—Pd1	115.8 (3)	C9—C10—H10B	109.2
C7—P1—Pd1	108.5 (3)	H10A—C10—H10B	107.9
C6—C1—C2	109.2 (7)	C10—C11—C12	111.6 (8)
C6—C1—P1	112.5 (6)	C10—C11—H11A	109.3
C2—C1—P1	116.7 (5)	C12—C11—H11A	109.3
C6—C1—H1	105.9	C10—C11—H11B	109.3
C2—C1—H1	105.9	C12—C11—H11B	109.3
P1—C1—H1	105.9	H11A—C11—H11B	108.0
C3—C2—C1	109.1 (7)	C11—C12—C7	110.5 (7)
C3—C2—H2A	109.9	C11—C12—H12A	109.6
C1—C2—H2A	109.9	C7—C12—H12A	109.6
C3—C2—H2B	109.9	C11—C12—H12B	109.6
C1—C2—H2B	109.9	C7—C12—H12B	109.6
H2A—C2—H2B	108.3	H12A—C12—H12B	108.1
C4—C3—C2	111.7 (7)	C14—C13—C18	120.4 (9)
C4—C3—H3A	109.3	C14—C13—H13	119.8
C2—C3—H3A	109.3	C18—C13—H13	119.8
C4—C3—H3B	109.3	C15—C14—C13	121.4 (9)
C2—C3—H3B	109.3	C15—C14—H14	119.3
H3A—C3—H3B	107.9	C13—C14—H14	119.3
C3—C4—C5	112.5 (7)	C14—C15—C16	119.8 (10)
C3—C4—H4A	109.1	C14—C15—H15	120.1
C5—C4—H4A	109.1	C16—C15—H15	120.1
C3—C4—H4B	109.1	C15—C16—C17	120.4 (10)
C5—C4—H4B	109.1	C15—C16—H16	119.8
H4A—C4—H4B	107.8	C17—C16—H16	119.8
C4—C5—C6	109.5 (7)	C16—C17—C18	120.9 (9)
C4—C5—H5A	109.8	C16—C17—H17	119.6
C6—C5—H5A	109.8	C18—C17—H17	119.6
C4—C5—H5B	109.8	C17—C18—C13	117.0 (8)
C6—C5—H5B	109.8	C17—C18—C19	121.4 (8)
H5A—C5—H5B	108.2	C13—C18—C19	121.4 (8)
C1—C6—C5	110.0 (7)	C20—C19—C24	118.2 (8)
C1—C6—H6A	109.7	C20—C19—C18	116.4 (7)
C5—C6—H6A	109.7	C24—C19—C18	125.4 (7)
C1—C6—H6B	109.7	C21—C20—C19	122.8 (9)
C5—C6—H6B	109.7	C21—C20—H20	118.6
H6A—C6—H6B	108.2	C19—C20—H20	118.6
C8—C7—C12	109.9 (7)	C22—C21—C20	119.2 (8)
C8—C7—P1	117.0 (6)	C22—C21—H21	120.4
C12—C7—P1	113.5 (5)	C20—C21—H21	120.4
C8—C7—H7	105.1	C21—C22—C23	120.5 (9)
C12—C7—H7	105.1	C21—C22—H22	119.8
P1—C7—H7	105.1	C23—C22—H22	119.8
C9—C8—C7	111.9 (8)	C24—C23—C22	121.3 (9)
C9—C8—H8A	109.2	C24—C23—H23	119.3
C7—C8—H8A	109.2	C22—C23—H23	119.3
C9—C8—H8B	109.2	C23—C24—C19	117.9 (7)
C7—C8—H8B	109.2	C23—C24—P1	117.6 (6)
H8A—C8—H8B	107.9	C19—C24—P1	124.5 (6)
			
P1^i^—Pd1—P1—C24	103 (35)	C9—C10—C11—C12	54.5 (12)
Br1^i^—Pd1—P1—C24	120.1 (3)	C10—C11—C12—C7	−57.0 (11)
Br1—Pd1—P1—C24	−59.9 (3)	C8—C7—C12—C11	57.1 (10)
P1^i^—Pd1—P1—C1	−20 (33)	P1—C7—C12—C11	−169.7 (7)
Br1^i^—Pd1—P1—C1	−2.1 (3)	C18—C13—C14—C15	0.5 (15)
Br1—Pd1—P1—C1	177.9 (3)	C13—C14—C15—C16	−1.5 (16)
P1^i^—Pd1—P1—C7	−142 (33)	C14—C15—C16—C17	−0.2 (16)
Br1^i^—Pd1—P1—C7	−124.4 (3)	C15—C16—C17—C18	2.9 (15)
Br1—Pd1—P1—C7	55.6 (3)	C16—C17—C18—C13	−3.7 (14)
C24—P1—C1—C6	168.1 (6)	C16—C17—C18—C19	−178.3 (9)
C7—P1—C1—C6	55.8 (6)	C14—C13—C18—C17	2.1 (13)
Pd1—P1—C1—C6	−66.5 (6)	C14—C13—C18—C19	176.6 (8)
C24—P1—C1—C2	40.7 (7)	C17—C18—C19—C20	57.9 (11)
C7—P1—C1—C2	−71.6 (7)	C13—C18—C19—C20	−116.4 (9)
Pd1—P1—C1—C2	166.1 (5)	C17—C18—C19—C24	−123.8 (9)
C6—C1—C2—C3	59.6 (9)	C13—C18—C19—C24	61.9 (12)
P1—C1—C2—C3	−171.5 (6)	C24—C19—C20—C21	−2.3 (13)
C1—C2—C3—C4	−55.9 (10)	C18—C19—C20—C21	176.1 (8)
C2—C3—C4—C5	54.7 (11)	C19—C20—C21—C22	1.9 (14)
C3—C4—C5—C6	−55.3 (10)	C20—C21—C22—C23	−1.4 (14)
C2—C1—C6—C5	−61.9 (9)	C21—C22—C23—C24	1.5 (14)
P1—C1—C6—C5	166.9 (6)	C22—C23—C24—C19	−1.8 (12)
C4—C5—C6—C1	58.9 (9)	C22—C23—C24—P1	−179.1 (7)
C24—P1—C7—C8	−64.7 (7)	C20—C19—C24—C23	2.2 (12)
C1—P1—C7—C8	48.4 (7)	C18—C19—C24—C23	−176.0 (8)
Pd1—P1—C7—C8	175.1 (6)	C20—C19—C24—P1	179.2 (6)
C24—P1—C7—C12	165.6 (6)	C18—C19—C24—P1	1.0 (12)
C1—P1—C7—C12	−81.3 (7)	C1—P1—C24—C23	−139.6 (6)
Pd1—P1—C7—C12	45.3 (7)	C7—P1—C24—C23	−24.7 (7)
C12—C7—C8—C9	−56.0 (10)	Pd1—P1—C24—C23	93.0 (6)
P1—C7—C8—C9	172.6 (7)	C1—P1—C24—C19	43.4 (8)
C7—C8—C9—C10	54.0 (12)	C7—P1—C24—C19	158.3 (7)
C8—C9—C10—C11	−52.8 (13)	Pd1—P1—C24—C19	−84.1 (7)

Symmetry codes: (i) −*x*+2, −*y*+1, −*z*+1.

## References

[bb1] Barder, T. E., Walker, S. D., Martinelli, J. R. & Buchwald, S. L. (2005). *J. Am. Chem. Soc.* **127**, 4685–4696. 10.1021/ja042491j 15796535

[bb2] Bruker (2004). *SMART* and *SAINT* Bruker AXS Inc., Madison, Wisconsin, USA.

[bb3] Christmann, U., Pantazis, D. A., Benet-Buchholz, J., McGrady, J. E., Maseras, F. & Vilar, R. (2006). *J. Am. Chem. Soc.* **128**, 6376–6390. 10.1021/ja057825z 16683802

[bb4] Sheldrick, G. M. (1996). *SADABS* University of Göttingen, Germany.

[bb5] Sheldrick, G. M. (2008). *Acta Cryst.* A**64**, 112–122. 10.1107/S0108767307043930 18156677

[bb6] Spek, A. L. (2003). *J. Appl. Cryst.* **36**, 7–13.

[bb7] Stark, J. L. & Whitmire, K. H. (1997). *Acta Cryst.* C**53**, IUC9700007.

[bb8] Tomori, H., Fox, J. M. & Buchwald, S. L. (2000). *J. Org. Chem.* **65**, 5334–5341. 10.1021/jo000691h 10993363

[bb9] Tsuji, J. (1995). *Palladium Reagents and Catalysts* Chichester: Wiley.

[bb10] Xu, C., Gong, J. F. & Wu, Y. J. (2007). *Tetrahedron Lett.* **48**, 1619–1623.

